# Rapid Identification of Potential Drugs for Diabetic Nephropathy Using Whole-Genome Expression Profiles of Glomeruli

**DOI:** 10.1155/2016/1634730

**Published:** 2016-03-16

**Authors:** Jingsong Shi, Song Jiang, Dandan Qiu, Weibo Le, Xiao Wang, Yinhui Lu, Zhihong Liu

**Affiliations:** National Clinical Research Center of Kidney Diseases, Research Institute of Nephrology, Jinling Hospital, Nanjing University School of Medicine, Nanjing 210016, China

## Abstract

*Objective*. To investigate potential drugs for diabetic nephropathy (DN) using whole-genome expression profiles and the Connectivity Map (CMAP).* Methodology*. Eighteen Chinese Han DN patients and six normal controls were included in this study. Whole-genome expression profiles of microdissected glomeruli were measured using the Affymetrix human U133 plus 2.0 chip. Differentially expressed genes (DEGs) between late stage and early stage DN samples and the CMAP database were used to identify potential drugs for DN using bioinformatics methods.* Results*. (1) A total of 1065 DEGs (FDR < 0.05 and fold change > 1.5) were found in late stage DN patients compared with early stage DN patients. (2) Piperlongumine, 15d-PGJ2 (15-delta prostaglandin J2), vorinostat, and trichostatin A were predicted to be the most promising potential drugs for DN, acting as NF-*κ*B inhibitors, histone deacetylase inhibitors (HDACIs), PI3K pathway inhibitors, or PPAR*γ* agonists, respectively.* Conclusion*. Using whole-genome expression profiles and the CMAP database, we rapidly predicted potential DN drugs, and therapeutic potential was confirmed by previously published studies. Animal experiments and clinical trials are needed to confirm both the safety and efficacy of these drugs in the treatment of DN.

## 1. Introduction

Diabetic nephropathy (DN), which is clinically characterized by proteinuria and morphological and ultrastructural changes in the kidney, is a serious complication of diabetes mellitus and is a major cause of end-stage renal disease worldwide. DN is a multifactorial progressive disease with complex pathogenesis, involving hyperglycemia, advanced glycation end products (AGEs), hemodynamic disorder, metabolic abnormalities, inflammatory factors, and oxidative stress [1]. Although our knowledge of DN is continuously increasing, no treatment strategies specifically target the pathogenesis of DN beyond controlling glucose levels, blood lipid levels, and high blood pressure [2]. As a result, the prognosis for most DN patients is poor, especially for those in late stages of the disease. The identification of potential drugs targeting the molecular pathogenesis of DN is critical for improving the prognosis and survival of patients with DN.

Whole-genome expression profiling is the simultaneous measurement of the expression of thousands of genes by microarray technology (or RNA-Seq) to create a global picture of tissue or cellular function. Comparing the whole-genome expression profiles of tissues (or cells) under physiologic and pathologic conditions may reveal the pathogenesis of DN. In addition to identifying differentially expressed and coexpressed genes, from which one can generate new hypotheses about the molecular mechanism of complex diseases, whole-genome expression data are also used to identify therapeutic drugs. The Connectivity Map (CMAP) database is a collection of genome-wide transcriptional expression data from cultured human cells treated with bioactive small molecules [3]. Lamb et al. findings showed that genomic signatures in the CMAP database can be used to identify potential new therapeutics, and signatures are often conserved across diverse cell types [3]. Therefore, the CMAP database can be used with whole-genome expression profiles to identify potential drugs for DN in the glomeruli of patients. Using the CMAP database, Zhong et al. predicted that the combination of an angiotensin-converting enzyme (ACE) inhibitor and a histone deacetylase inhibitor would maximally reverse the disease-associated expression of genes in a mouse model of HIV-associated nephropathy (Tg26 mice), and the renoprotective effect of the combined use of these inhibitors was proven in Tg26 mice [4]. It is feasible to utilize gene expression profiles of tissues under normal and physiopathological conditions to investigate potential therapeutic drugs based on bioinformatics methods. However, this kind of therapeutic drug identification in DN research is lacking. In our study, we utilized the gene expression profiles of microdissected glomeruli from DN patients and explored potential therapeutic drugs using in silico screening approaches.

## 2. Materials and Methods

### 2.1. Patients

The clinical study used a cross-sectional design. The control and DN kidney samples were obtained from leftover portions of diagnostic kidney biopsies. For the kidney biopsies, informed consent was obtained from the donors and patients. All of the participants provided written informed consent. The institutional review board of Jinling Hospital specifically approved this study [5].

A total of 18 DN patients diagnosed by renal biopsy were enrolled in the study. The baseline clinical characteristics of the DN patients are listed in Table 1.

For each biopsy specimen, light microscopy, immunofluorescence, and electron microscopy were routinely performed. Sections for light microscopy were stained with hematoxylin eosin, periodic acid-Schiff, Masson's trichrome, and periodic acid methenamine silver. All of the patients were categorized based on the pathologic classification of the Renal Pathology Society [5]. The glomerular classifications were as follows: class I, glomerular basement membrane thickening; class IIa, mild mesangial expansion; class IIb, severe mesangial expansion; class III, nodular sclerosis; and class IV, global glomerulosclerosis in >50% of glomeruli. Interstitial fibrosis and tubular atrophy (IFTA) were scored as follows: 0, absent; 1, <25%; 2, 25–50%; and 3, >50% of the total area. Interstitial inflammation was scored as follows: 0, absent; 1, inflammation only in relation to IFTA; and 2, inflammation in areas without IFTA. Arteriolar hyalinosis was scored as follows: 0, absent; 1, at least one area of arteriolar hyalinosis; and 2, more than one area of arteriolar hyalinosis. Arteriosclerosis was scored as follows: NA, absence of large vessels; 0, no intimal thickening; 1, intimal thickening less than thickness of media; and 2, intimal thickening greater than thickness of media. All of the specimens were scored by the same pathologist (Dr. Feng Xu) who was blinded to the clinical findings. In order to assess the reliability and reproducibility of the classification, biopsy slides were scored independently by another pathologist (Dr. Dandan Liang). The pathologic characteristics of the DN patients are listed in Table 1.

The DN patients were divided into 2 groups according to the following criteria: early stage DN group (*N* = 6), glomeruli isolated from the renal tissue of early stage DN patients who were identified with eGFR > 90 mL/min; late stage DN group (*N* = 12), glomeruli isolated from the renal tissue of late stage DN patients with eGFR between 15 mL/min and 60 mL/min. Control samples (*N* = 6) were obtained from living donor kidney biopsies. Control subjects were defined as having an eGFR of more than 90 mL/min, the absence of proteinuria, normal serum creatinine, and BUN.

### 2.2. Tissue Handling and Microdissection

Tissues placed in RNALater (SIGMA, St. Louis, MO, USA) were manually microdissected at 4°C for glomeruli. In general, 10 glomeruli were collected from each biopsy tissue and were placed into cold RNeasy lysis buffer solution (Qiagen, Valencia, CA, USA).

### 2.3. RNA Extraction and Amplification

Dissected glomeruli were homogenized, and RNA was prepared using RNeasy mini columns (Qiagen, Valencia, CA, USA), according to the manufacturer's instructions. RNA quality and quantity were determined using the Laboratory-on-Chip Total RNA Pico Kit Agilent Bioanalyzer. Samples without evidence of degradation were further amplified using the Ovation RNA amplification system kit (NuGEN, San Carlos, CA, USA).

### 2.4. Affymetrix Microarray Data and Preprocessing

The Affymetrix microarray platform (Human U133 Plus 2.0) was used to produce the whole-genome gene expression profile data. Quality control and data processing were performed using R [6] and Bioconductor [7]. The CEL source files were processed into expression estimates, and background correction and quartile data normalization were performed using the RMA (robust multiarray average) algorithm [8].

### 2.5. Screening of Differentially Expressed Genes (DEGs)

The limma package [9] in R language was used to screen DEGs by pairwise comparison between groups. The statistical method implemented in the limma package is based on an approach called linear models. We used the method proposed by Benjamini-Hochberg (BH) for multiple testing correction. The adjusted *P* values were the false discovery rates (FDR). The threshold criterion is a combination of FDR < 0.05 and fold change > 1.5. The DEGs between late stage and early stage DN samples were chosen for DN drug identification.

### 2.6. Validation of Microarray Expression Data

The relative mRNA levels of 10 genes were validated in new selected glomerular samples. The clinical and pathologic characteristics of these DN patients are listed in Table 2. The processes used for patient screening, tissue handling, microdissection, and total RNA were performed as previously described. The mRNA levels of the target genes were analyzed by quantitative real-time RT-PCR (qRT-PCR) using the Applied Biosystems*™* 7900HT Fast Real-Time PCR System (Thermo Fisher Scientific, Waltham, MA, USA). The qRT-PCR results were normalized to 18S ribosomal RNA using the 2^−ΔΔCT^ method [10], and significance was set to *P* < 0.05.

### 2.7. Drug Identification Using a Kolmogorov-Smirnov (KS) Statistic Algorithm

From the CMAP database (http://www.broadinstitute.org/cmap/), we downloaded the ranked lists of probe tables. This table is freely available for download and provides the ranks of all genes in the form of probes based on the change of gene expression after approximately 6000 drug perturbation experiments. The table contains 22283 probes, which have 22277 common probes with Human U133 Plus 2.0. Finally, we obtained a table containing 24 samples and 22277 probes. With the limma package in R language, we obtained the 1000 largest changing probes between the late and early stage DN samples, and the upregulated probes and the downregulated probes were saved in GRP files. The CMAP website provides a KS statistic algorithm; we uploaded the GRP files as the query gene signature, which was then compared to each rank-ordered list to determine whether upregulated query genes appeared near the top of the list and downregulated query genes near the bottom (positive connectivity) or vice versa (negative connectivity), yielding a connectivity score ranging from 1 to −1. A high negative connectivity score indicated that the corresponding perturbagen reversed the expression of the query signature and might have the potential to treat DN.

### 2.8. Drug Identification Using a Matching Algorithm [4]

The IDs of the probe table downloaded from the CMAP database were converted to Entrez gene symbols using the Affymetrix lookup table associated with the platform. If more than one probe ID corresponded to the same gene, the gene rank was condensed to the median of the probe ranks. Then, we extracted the 500 top and bottom DEGs. Potential DN drugs should reverse the DEGs of DN; genes upregulated in DN should be downregulated by a potential DN drug and vice versa. The DEGs (FDR < 0.05 and fold change > 1.5) between the late and early groups were used to calculate the reversing scores using formula (1). The perturbagen with the highest reversing scores might have the potential to treat DN. Consider

(1) Scoredi=up∩downdi+down∩updi−up∩updi+down∩downdi,

where Score_*di*_ indicates the reversing score for a drug in *i*th experiment in the CMAP database; ∩ indicates the intersection between two sets; up indicates a list of upregulated genes during disease; down indicates a list of downregulated genes during disease; up_*di*_ indicates genes upregulated by a drug in the *i*th experiment in the CMAP database; and down_*di*_ indicates genes downregulated by a drug in the *i*th experiment in the CMAP database.

### 2.9. Functional Enrichment Analysis

The functional enrichment analysis of the screened DEGs and the genes reversed by potential drugs was performed via the GeneAnswers package in R language [11]. The GeneAnswers package functionally categorizes genes based on Fisher's exact test with annotation libraries of the gene ontology (GO) and the Kyoto Encyclopedia of Genes and Genomes (KEGG).

## 3. Results

### 3.1. DEGs in the Glomeruli of DN Patients

We separately compared the samples in the 2 stages of DN with the controls. A total of 105 DEGs were identified between the early stage DN samples and the controls, including 54 upregulated genes and 51 downregulated genes; 2572 DEGs were identified between the late stage DN samples and the controls, including 1626 upregulated genes and 946 downregulated genes.

Only 105 DEGs were identified between the early stage DN samples and the controls, and the enriched GO categories were primarily involved in “response to stimulus.” These results were in accordance with the mild pathological changes in the glomeruli from early stage DN patients.

**Figure 1:**
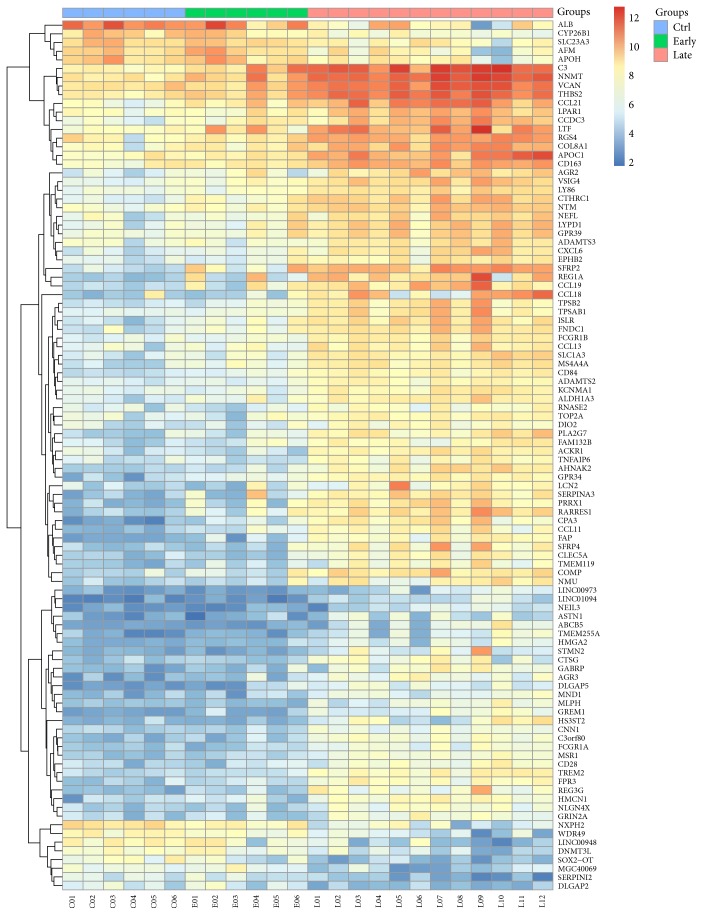
Heat map of the top 100 DEGs between glomeruli in the late and early stages of DN.

**Figure 2:**
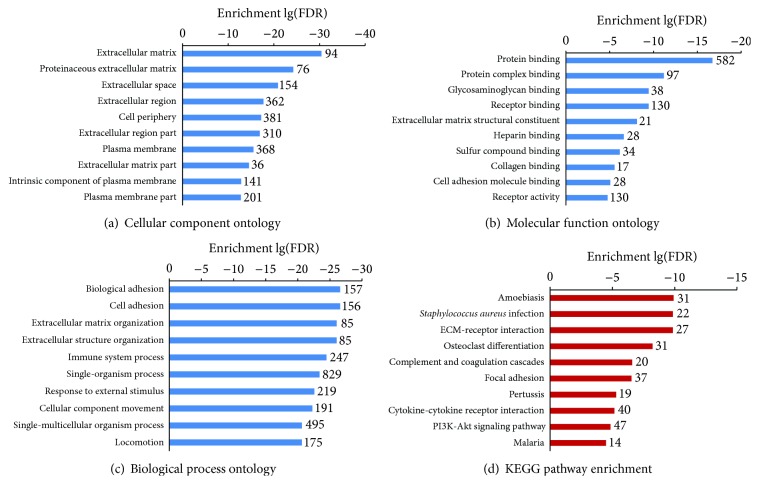
GO category (cellular component, molecular function, and biological process) and KEGG pathways enriched in the DEGs between glomeruli in the late and early stages of DN.

In contrast, 1065 DEGs were identified between the late and early stages of DN samples, including 815 upregulated genes and 250 downregulated genes. The heat map in Figure 1 shows the expression levels of the top 100 regulated genes across the 24 samples. As shown in Figure 2, the most enriched GO categories of the DEGs were “extracellular matrix,” “protein binding,” “cell adhesion,” and “immune system process.” The most enriched KEGG pathways were “ECM-receptor interaction,” “complement and coagulation cascades,” “focal adhesion,” “cytokine-cytokine receptor interaction,” and “PI3K-Akt signaling pathway.” These GO categories and KEGG pathways are closely related to DN progression [12, 13].

**Figure 3:**
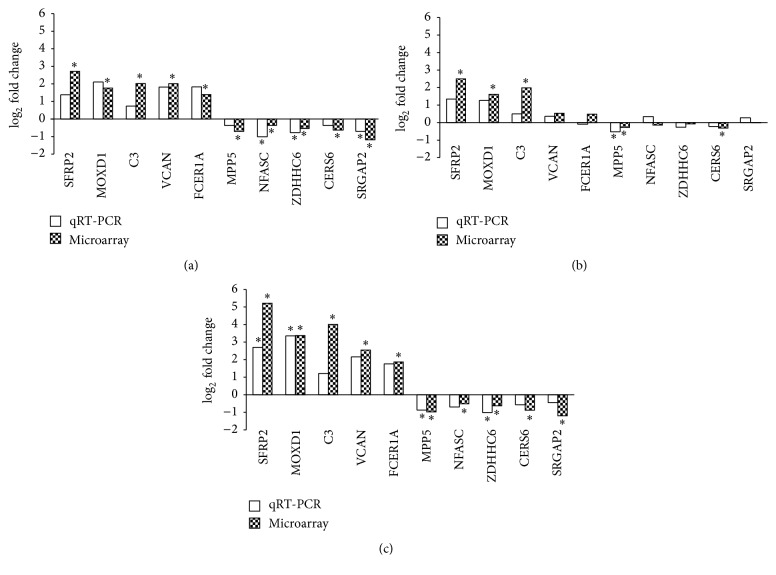
qRT-PCR confirmation. qRT-PCR was performed to confirm the direction of the fold change in expression (shown as log_2_ fold change). The gene expression changes determined by qRT-PCR were compared with those obtained from the microarray analysis of late versus early stage DN (a), early stage DN versus Ctrl (b), and late stage DN versus Ctrl (c). ^*∗*^ *P* < 0.05.

As shown in Figure 3, qRT-PCR analysis was performed to confirm the degree and direction of the expression changes in 10 genes. All 10 genes assayed by qRT-PCR were found to be significantly differentially expressed in the microarray analysis between the late and early stage DN samples. As determined by qRT-PCR analysis, 10 out of the 10 selected genes demonstrated a change in expression in the same direction (i.e., up- or downregulated) (Figure 3(a)). Similarly, the direction of the change in gene expression determined by qRT-PCR analysis agreed with the directions obtained for the genes that were found to be significantly differentially expressed by microarray analysis between the early stage DN samples and the controls (5 out of 5 genes, Figure 3(b)) and between the late stage DN samples and the controls (10 out of 10 genes, Figure 3(c)).

### 3.2. Potential DN Drugs Predicted by the KS Statistic Algorithm

To explore the potential drugs targeting the molecular mechanisms of DN, we used the DEGs between the late and early stages of DN. These genes were enriched for their specific contribution to nephropathy because genes that are differentially regulated in human diabetes per se, in the absence of nephropathy, were excluded by this strategy. After uploading 812 upregulated and 188 downregulated probe IDs to the CMAP database, the top 20 drug perturbations that most strongly reversed the DRGs are listed in Table 3. Among these drugs, MG-132 [14] and MG-262 [15] are proteasome inhibitors, piperlongumine inhibits PI3K/Akt/mTOR signaling [16] and NF-*κ*B activity [17], 15d-PGJ2 (15-delta prostaglandin J2) activates PPAR*γ* [18], and vorinostat and trichostatin A are histone deacetylase inhibitors (HDACIs).

### 3.3. Potential DN Drugs Predicted by the Matching Algorithm

**Figure 4:**
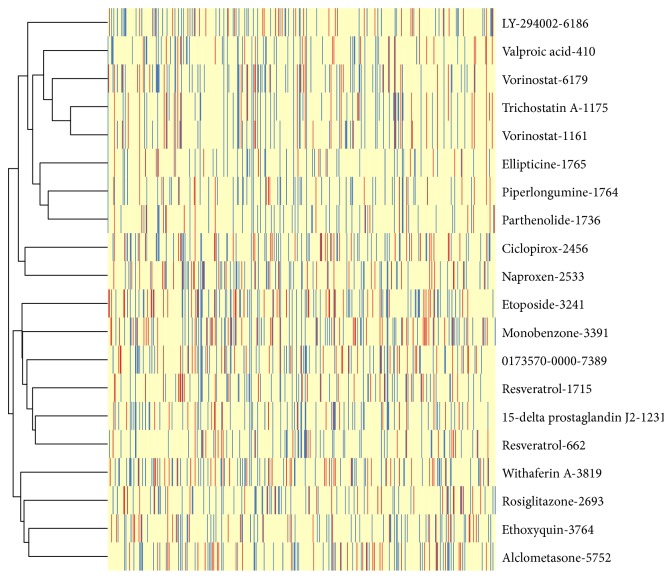
The heat map and hierarchical cluster of effects of the top 20 drug perturbations. Each row indicates a drug perturbation, and each column represents a gene. The blue vertical bars in the heat map indicate that the gene was reversed by the corresponding perturbagen, whereas the red bars indicate that the gene was aggravated by the corresponding perturbagen. Drug perturbations having similar effects were clustered together.

We further utilized a matching algorithm and the DEGs between early and late stage DN samples to calculate the reversing score for each drug in the CMAP database. The top 20 drug perturbations that had the highest scores are listed in Table 4. We clustered the drugs by the similarity of reversed DEGs, and the drugs with similar pharmacological characteristics were clustered together (Figure 4). For example, HDACIs, including vorinostat, trichostatin A, and valproic acid, were clustered together. Among these drugs, piperlongumine, 15d-PGJ2, vorinostat, and trichostatin A were also identified by the KS statistic algorithm. Valproic acid and parthenolide are also histone deacetylase inhibitors [19], resveratrol inhibits cAMP phosphodiesterase [20], and LY-294002 inhibits PI3K [21].

### 3.4. Functional Enrichment Analysis of the Drug-Reversed Genes

**Figure 5:**
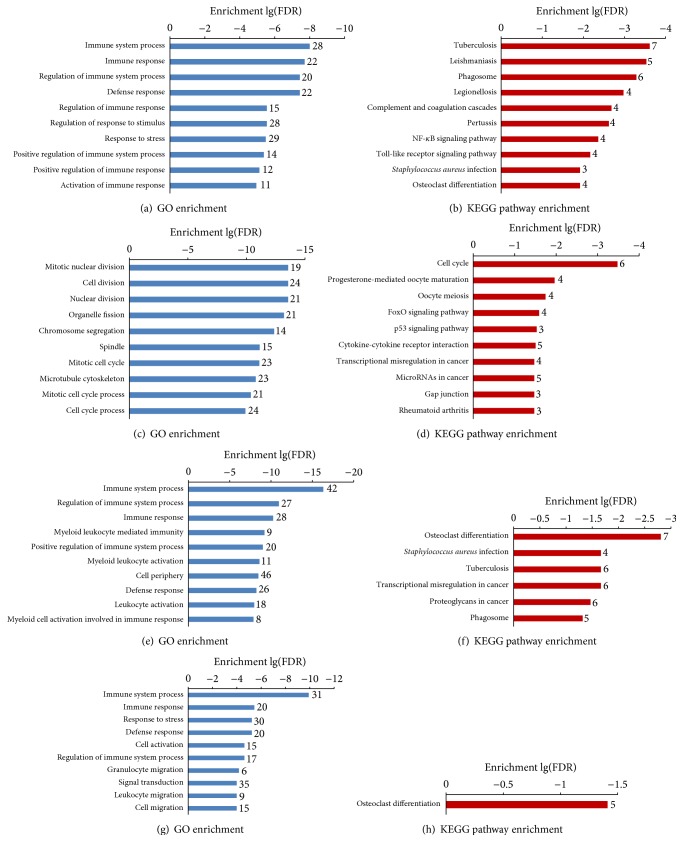
GO categories and KEGG pathways enriched in the DEGs that were reversed by the drug perturbation. (a), (b) GO categories and KEGG pathways enriched in the DEGs that were reversed by piperlongumine; (c), (d) GO categories and KEGG pathways enriched in the DEGs that were reversed by 15d-PGJ2; (e), (f) GO categories and KEGG pathways enriched in the DEGs that were reversed by vorinostat; (g), (h) GO categories and KEGG pathways enriched in the DEGs that were reversed by trichostatin A.

Piperlongumine, 15d-PGJ2, vorinostat, and trichostatin A were identified by both algorithms. The genes that could be reversed by these drugs are shown in Table 5. To indicate the target molecular mechanisms of these drugs, we conducted a functional analysis of these potentially reversed genes in the glomeruli of DN patients (Figure 5). The genes reversed by piperlongumine are mostly involved in the “immune response,” “response to stimulus,” “complement and coagulation cascades,” “NF-*κ*B signaling pathway,” and “Toll-like receptor signaling pathway.” The genes reversed by 15d-PGJ2 are primarily involved in “cell division,” “FoxO signaling pathway,” and “cytokine-cytokine receptor interaction,” and the genes reversed by vorinostat are mostly involved in the “immune response,” “response to stimulus,” “signal transduction,” and “osteoclast differentiation.” The genes reversed by trichostatin A are mostly involved in the “immune response,” “response to stress,” “signal transduction,” “cell migration,” and “osteoclast differentiation.”

## 4. Discussion

Current clinical strategies to treat DN focus on the intensification of glycemic control and the control of blood pressure and blood fat. Renoprotective drugs based on the molecular pathogenesis of DN are unavailable because the molecular pathogenesis of DN is complicated. Recently, high-throughput transcriptome technology has been used to explore the molecular pathogenesis of complex diseases [22, 23], and drug screening methods based on transcriptome data have attracted increasing attention [4, 24].

The most common kidney lesions in people with diabetes are those that affect the glomeruli, and DN is characterized by morphological and ultrastructural changes in the kidney, including expansion of the molecular matrix and loss of the charge barrier on the glomerular basement membrane [25]. The gene expression profiles of glomeruli microdissected from DN biopsy samples will provide an excellent opportunity to explore the molecular mechanisms of this complex disease and to identify potential drugs.

In this study, we obtained the whole-genome transcriptome profiles of glomeruli from DN patients and normal controls. The glomeruli contain podocytes, endothelial cells, and mesangial cells, whereas the CMAP database was built upon four types of cultured human cell lines [3]. Because the signatures of drugs are often conserved across diverse cell types [3], we can utilize the CMAP database to identify potential drugs for DN.

There were only 105 DEGs between early stage DN samples and controls, mainly involving “response to stimulus.” These results indicated that there was minimal gene expression change involving molecular pathogenesis in the early stage of DN. The DEGs between the late and early stages of DN samples were related to “extracellular matrix,” “cell adhesion,” “immune system process,” “ECM-receptor interaction,” “complement and coagulation cascades,” “cytokine-cytokine receptor interaction,” and “PI3K-Akt signaling pathway” (Figure 2). These functional categories and pathways have been widely related to the pathogenesis of DN [13]. Coexpression network analysis and association analysis indicated that some DEGs between DN samples and controls exhibit no correlation with the progression or prognosis of DN (data not shown). These genes, which may be differentially regulated in human diabetes per se, were excluded from the comparison of the late and early stage DN samples. Therefore, it is more suitable to use the DEGs between the late and early stages to identify potential therapeutic drugs for DN treatment.

A KS statistic algorithm and a matching algorithm were applied in this study. However, using either algorithm, some drugs among the top 20 are unlikely to have renal protection on the basis of prior clinical and pharmacological knowledge. Therefore, the results based on the two different algorithms were combined to enhance the reliability of the potential therapeutic drugs, which provided a good foundation for the in vitro and in vivo studies.

Piperlongumine, 15d-PGJ2, vorinostat, and trichostatin A were identified by both algorithms. The molecular mechanisms of the 4 drugs include inhibition of NF-*κ*B activity, histone deacetylase (HDAC) activity, PI3K-Akt signaling pathway, and the activation of PPAR*γ*. The transcription factor NF-*κ*B is induced by various cell stress-associated stimuli, including growth factors, vasoactive agents, cytokines, and oxidative stress. NF-*κ*B in turn controls the regulation of genes encoding proteins involved in immune and inflammatory responses. The activation and nuclear translocation of NF-*κ*B in human DN have been demonstrated in the intrinsic cells of the kidney [26]. Upregulation of HDACs has been reported in the kidneys of patients with DN as well as in type 1 and type 2 in vivo animal models of diabetes [27]. HDACIs have anti-inflammatory and antifibrotic effects in the kidney and may prove to be a novel class of agents in the treatment of diabetic nephropathy [27]. The PI3K-Akt signaling pathway is directly related to cellular proliferation, migration, differentiation, and survival. There are many known factors that enhance the PI3K-Akt pathway, including insulin, EGF, and IGF-1. In rat mesangial cells and db/db mice, high-glucose decreased the expression of MNSOD via the PI3K-Akt signaling pathway and further aggravated oxidative stress [28]. The nuclear receptor PPAR*γ* is located in all three types of glomerular cells, with prominent expression in podocytes. PPAR*γ* agonists, which have emerged as promising candidates for treating DN, are effective in delaying and even preventing disease progression.

Piperlongumine can inhibit both NF-*κ*B activation and the PI3K-Akt signaling pathway [16, 17] and increase mRNA levels of PPAR*γ*2 [29]. Piperlongumine is a main component of the root of* Piper longum*, a plant used by some Indian tribes to treat diabetes, digestive disorders, and obesity [30]. In streptozotocin- (STZ-) induced diabetic rats,* Piper longum* root aqueous extract can significantly decrease fasting blood glucose levels and protect liver and kidney function [30].

15d-PGJ2 is the endogenous ligand of PPAR*γ* and can regulate metabolism of adipose tissue and restrain insulin resistance [18]. In rat mesangial cells, 15d-PGJ2 significantly decreased alpha-smooth muscle actin (*α*-SMA) expression, a marker of mesangial cell dedifferentiation. In mouse mesangial cells, 15d-PGJ2 repressed TGF-*β*1-mediated *α*-SMA, fibronectin, and plasminogen activator inhibitor-1 expression; induced HGF expression; and attenuated Smad nuclear translocation in response to TGF-*β*1 stimulation [31]. In rat renal interstitial fibroblasts, 15d-PGJ2 inhibited TGF-*β*1-induced renal fibroblast activation, CTGF expression, and ECM synthesis through abrogating the TGF-*β*1/Smad signaling pathway [32].

Vorinostat and trichostatin A belong to the same group of HDACIs based on their chemical structures (hydroxamic acid). They are broad inhibitors of HDAC activity and inhibit class I and class II enzymes [33]. Vorinostat is FDA approved for use against refractory cutaneous T cell lymphoma [34, 35]. HDACIs have beneficial effects in diabetic nephropathy. In cultured proximal tubule cells, vorinostat treatment reduced EGFR protein and mRNA and attenuated cellular proliferation [36]. Daily treatment of diabetic rats with vorinostat for 4 weeks blunted renal growth and glomerular hypertrophy [36]. In STZ-induced diabetic mice, long-term administration of vorinostat decreased albuminuria, mesangial collagen IV deposition, and oxidative-nitrosative stress through an eNOS-dependent mechanism [37]. In STZ-induced diabetic rats, trichostatin A prevented extracellular matrix accumulation and epithelial-to-mesenchymal transition in diabetic kidney [38].

In addition to these four drugs, we have identified potential therapeutic drugs to treat DN. Proteasome inhibitors, including MG-132 and MG-262, have anti-inflammatory and antifibrotic effects [15]. MG132 alleviates kidney damage by inhibiting Smad7 ubiquitin degradation, SnoN degradation, and TGF-*β* activation in STZ-induced DN rats [39, 40]. Valproic acid, another histone deacetylase inhibitor, has beneficial effects on proteinuria, glomerulosclerosis, and renal inflammation in Adriamycin-induced nephropathic mice [41]. LY-294002, a PI3K inhibitor, prevented the quantitative and distributional changes of CD2AP induced by high-glucose and advanced glycosylation end products in mouse podocytes [42]. In db/db mice, LY-294002 decreased the levels of phosphorylated Akt and phosphorylated FoxO3a, increased the level of MnSOD expression, and further decreased oxidative stress [28]. Resveratrol, a phosphodiesterase inhibitor, improved diabetic nephropathy in several animal models of types 1 and 2 diabetes through its antioxidative effects resulting from direct radical scavenging or modulation of antioxidant enzymes [43].

In summary, this study utilized the gene expression profiles of glomeruli from DN patients to identify potential drugs for DN using the CMAP database and bioinformatics methods. Four drugs were identified by two different algorithms, and therapeutic potential was shown to be promising by literature analysis. Our study provides useful information for further animal experiments and clinical trials to confirm both the safety and efficacy of these drugs.

## Supplementary Material

S1. DEGs between early stage DN samples and the normal controls.
S2. DEGs between late stage DN samples and the normal controls.
S3. DEGs between early and late stage DN samples.

## Figures and Tables

**Table 1:** The baseline clinical and pathologic characteristics of DN patients.

	Early stage	Late stage	*P*
*n*	6	12	—
Age (years)	45.9 ± 6.4	51.0 ± 6.8	0.103
Sex (female, %)	3 (50%)	3 (25%)	0.344
Ethnicity	Han	Han	—
BMI (kg/m^2^)	25.3 ± 1.5	24.9 ± 1.3	0.892
Serum creatinine (mg/dL)	0.63 ± 0.13	2.34 ± 0.74	0.000
eGFR (mL/min)	112.8 ± 8.1	32.9 ± 13.3	0.000
Proteinuria (g/24 h)	0.82 ± 0.47	5.18 ± 2.01	0.000
HbA1C (%)	8.4 ± 1.6	7.2 ± 1.9	0.146
BUN (mg/dL)	15.4 ± 4.8	35.8 ± 11.8	0.003
Glomerular lesions			0.000
Class I	3	0	
Class IIa	3	0	
Class IIb	0	0	
Class III	0	7	
Class IV	0	5	
IFTA			0.006
0	2	0	
1	4	2	
2	0	4	
3	0	6	
Interstitial inflammation			0.004
0	2	0	
1	3	1	
2	1	11	
Arteriolar hyalinosis			0.333
0	0	0	
1	1	0	
2	5	12	
Arteriosclerosis			0.504
NA	0	0	
0	2	1	
1	1	2	
** **2	3	9	

BMI: body mass index; eGFR: estimated glomerular filtration rate, calculated using the EPI-CKD formula; HbA1C: glycated hemoglobin; BUN: blood urea nitrogen. Values are presented as *n* or means ± SD. *P* values were obtained using the Wilcoxon rank sum test for continuous variables and Fisher's exact test for categorical variables.

**Table 2:** The baseline clinical and pathologic characteristics of DN patients for validation.

	Early stage	Late stage	*P*
*n*	5	4	—
Age (years)	43.1 ± 11.8	52.1 ± 7.3	0.226
Sex (female, %)	3 (60%)	2 (50%)	1
Ethnicity	Han	Han	—
BMI (kg/m^2^)	25.5 ± 4.7	22.9 ± 2.3	0.342
Serum creatinine (mg/dL)	0.65 ± 0.15	1.76 ± 0.54	0.003
eGFR (mL/min)	112.3 ± 4.3	42.1 ± 17.4	0.000
Proteinuria (g/24 h)	0.50 ± 0.23	4.43 ± 0.41	0.000
HbA1C (%)	6.5 ± 0.9	7.3 ± 1.0	0.285
BUN (mg/dL)	14.2 ± 2.7	30.0 ± 6.8	0.002
Glomerular lesions			0.016
Class I	1	0	
Class IIa	4	0	
Class IIb	0	0	
Class III	0	2	
Class IV	0	2	
IFTA			0.087
0	1	0	
1	4	1	
2	0	3	
3	0	0	
Interstitial inflammation			0.286
0	1	0	
1	4	2	
2	0	2	
Arteriolar hyalinosis			1
0	0	0	
1	0	0	
2	5	4	
Arteriosclerosis			1
NA	0	0	
0	1	0	
1	2	1	
2	2	3	

**Table 3:** Top 20 drug perturbations with high negative connectivity scores.

Rank	Instance ID	CMAP name	Dose	Cell	Score	Up	Down
1	1274	Bepridil	10 *µ*M	HL60	−1	−0.077	0.223
2	7063	MG-262	100 nM	MCF7	−0.96	−0.163	0.125
3	7345	Alcuronium chloride	5 *µ*M	MCF7	−0.953	−0.108	0.178
4	942	Prazosin	10 *µ*M	MCF7	−0.953	−0.111	0.175
5	1764	Piperlongumine	13 *µ*M	HL60	−0.94	−0.071	0.212
6	7022	Dyclonine	12 *µ*M	MCF7	−0.931	−0.104	0.175
7	3351	(±)-Catechin	14 *µ*M	MCF7	−0.923	−0.126	0.151
8	7017	Mesoridazine	7 *µ*M	MCF7	−0.922	−0.101	0.176
9	909	HC toxin	100 nM	MCF7	−0.921	−0.107	0.17
10	1694	Metformin	24 *µ*M	MCF7	−0.909	−0.102	0.17
11	1656	15d-PGJ2	10 *µ*M	MCF7	−0.901	−0.112	0.158
12	7020	Xylometazoline	14 *µ*M	MCF7	−0.898	−0.1	0.17
13	1058	Vorinostat	10 *µ*M	MCF7	−0.898	−0.12	0.15
14	7178	Tetrandrine	6 *µ*M	MCF7	−0.897	−0.151	0.118
15	1069	15d-PGJ2	10 *µ*M	MCF7	−0.895	−0.12	0.149
16	1140	MG-132	21 *µ*M	MCF7	−0.884	−0.122	0.143
17	1112	Trichostatin A	100 nM	MCF7	−0.882	−0.104	0.161
18	6936	Chlorpromazine	1 *µ*M	MCF7	−0.88	−0.115	0.149
19	5310	Puromycin	7 *µ*M	MCF7	−0.874	−0.111	0.151
20	5304	Moroxydine	19 *µ*M	MCF7	−0.874	−0.125	0.137

Instance: a treatment and control pair and the list of probe sets ordered by their extent of differential expression between this treatment and control pair; instance ID: the ID uniquely identifying each instance; CMAP name: the name given to a perturbagen; dose: perturbagen dose; cell: cell line; up: the up score, a value between +1 and −1 representing the absolute enrichment of an up tag list in a given instance; down: the down score, a value between +1 and −1 representing the absolute enrichment of a down tag list in a given instance; score: the connectivity score, a combination of the up score and the down score. A high negative connectivity score indicates that the corresponding perturbagen reversed the expression of the query signature.

**Table 4:** Top 20 drug perturbations with high reversing scores.

Perturbagen name-instance ID	Score	Reversed	Aggravated
Vorinostat-6179	46	75	29
15d-PGJ2-1231	43	65	22
Piperlongumine-1764	36	55	19
Resveratrol-662	34	54	20
Ciclopirox-2456	33	67	34
Resveratrol-1715	32	59	27
LY-294002-6186	32	63	31
Valproic acid-410	31	53	22
Trichostatin A-1175	31	59	28
Ethoxyquin-3764	31	57	26
0173570-0000-7389	31	68	37
Parthenolide-1736	30	47	17
Ellipticine-1765	30	46	16
Etoposide-3241	30	73	43
Withaferin A-3819	30	68	38
Vorinostat-1161	29	57	28
Naproxen-2533	29	63	34
Rosiglitazone-2693	29	55	26
Monobenzone-3391	29	74	45
Alclometasone-5752	29	63	34

Perturbagen name: the name given to a perturbagen; instance ID: the ID uniquely identifying each instance; score: the reversing score calculated using formula (1); reversed: number of genes in the query signature, the expression of these genes was reversed by the corresponding perturbagen; aggravated: number of genes in the query signature, the expression of these genes was aggravated by the corresponding perturbagen.

**Table 5:** DEGs reversed by drug perturbations.

Drugs	Direction	Genes
Piperlongumine	Decrease	CLEC5A, TNFAIP6, FCGR1B, C3, NEIL3, RNASE6, C3AR1, SAMSN1, CAPG, CD86, TREM1, IRF8, EVI2A, PBK, FCGR2A, RGS13, KCNN4, GPR183, TLR2, GINS2, CLEC7A, RRM2, RGS1, CD300A, CD14, CTSC, TMEM158, CDCA3, NCF2, SYK, IL1B, GINS1, SLC43A3, PSRC1, MYC, FILIP1L, MYO1F, PLAUR, PFKP, CCR1, HELLS, PLAU, PTPRE, MNDA, TNFAIP8, MAD2L1, CYP1B1, RASSF2, GALNT7, ECT2, CD300C, CDC45
	Increase	HPS5, EAF2, RND1

15d-PGJ2	Decrease	MLPH, CXCL6, TOP2A, LPAR1, GPR39, DLGAP5, HMGA2, SCG5, CXCL1, PTX3, INHBA, TNFRSF11A, COL5A2, TENM4, ASPM, KIF18B, CENPF, KIF20A, IL33, SYTL2, CCNB2, CCNA1, HJURP, CDC20, CDH11, LMNB1, NCAPH, MKI67, CDCA3, FST, TTK, NDP, GINS1, MTCL1, PSRC1, NEK2, HAS2, CDCA8, NPM3, ETV1, KIF2C, FAM110B, PRSS23, CDK1, PLAU, MALL, P2RY6, AURKB, BNC2, FJX1, CCNB1, TROAP, IGSF3, CYP1B1, ECT2, TK1, IRS1
	Increase	PRKCE, SLC16A10, ERVMER34-1, EAF2, RND1, EGF, ZNF804A, SERPINI2

Vorinostat	Decrease	CLEC5A, PLA2G7, CTSG, FCGR1A, FCGR1B, VCAN, LAIR1, BCAT1, HP, PTAFR, C3AR1, ADORA3, LILRB4, HGF, IGSF6, CCR2, NCF4, MS4A6A, CD33, MMP9, GNA15, EVI2A, TLR1, ATP8B4, CACNA2D3, LILRB1, KCNN4, GPR183, ANXA3, TLR2, CLEC7A, RRM2, RGS1, LMNB1, NINJ2, CD300A, CSF2RA, NCAPH, CD14, INHBE, LILRA2, SIGLEC9, LAT2, SYK, SLC38A1, SLC43A3, MYC, GLIPR1, PTENP1, MYO1F, PSTPIP1, PLAUR, CCR1, PLAU, DOK3, SELPLG, SASH3, P2RY6, VAV1, PTPRE, MNDA, SPI1, DEF6, CYBRD1, CXorf21, CYP1B1, RASSF2, DOK2
	Increase	ACOX2, EXPH5, PRKAR2B, GDPD3, MLXIPL, RND1, CTSV

Trichostatin A	Decrease	CLEC5A, FCGR1B, VCAN, LAIR1, RNASE6, C3AR1, HGF, IGSF6, NCF4, MS4A6A, SLC7A11, EVI2A, KIF20A, ATP8B4, CACNA2D3, RGS13, KCNN4, GPR183, ANXA3, CLEC7A, RRM2, RGS1, LMNB1, CD300A, CD14, INHBE, PYCARD, LILRA2, SYK, SLC38A1, SLC43A3, PIK3CG, MYC, FILIP1L, PSTPIP1, PLAUR, FOXM1, PLAU, SASH3, P2RY6, VAV1, MNDA, SPI1, CSF3R, CYBRD1, CXorf21, MICAL1, CYP1B1, RCC1, P2RX1, ARRB2, RASSF2, GALNT7, DOK2, FAM129A
	Increase	PRKAR2B, CTNNBIP1, RND1, CTSV
